# QTL Mapping of Intestinal Neutrophil Variation in Threespine Stickleback Reveals Possible Gene Targets Connecting Intestinal Inflammation and Systemic Health

**DOI:** 10.1534/g3.119.400685

**Published:** 2019-12-16

**Authors:** Emily A. Beck, Mark C. Currey, Clayton M. Small, William A. Cresko

**Affiliations:** Institute of Ecology and Evolution, University of Oregon, Eugene OR, 97403

**Keywords:** QTL mapping, neutrophil, inflammation, immunity, threespine stickleback, Genetics of Immunity

## Abstract

Selection, via host immunity, is often required to foster beneficial microbial symbionts and suppress deleterious pathogens. In animals, the host immune system is at the center of this relationship. Failed host immune system-microbial interactions can result in a persistent inflammatory response in which the immune system indiscriminately attacks resident microbes, and at times the host cells themselves, leading to diseases such as Ulcerative Colitis, Crohn’s Disease, and Psoriasis. Host genetic variation has been linked to both microbiome diversity and to severity of such inflammatory disease states in humans. However, the microbiome and inflammatory states manifest as quantitative traits, which encompass many genes interacting with one another and the environment. The mechanistic relationships among all of these interacting components are still not clear. Developing natural genetic models of host-microbe interactions is therefore fundamental to understanding the complex genetics of these and other diseases. Threespine stickleback (*Gasterosteus aculeatus*) fish are a tractable model for attacking this problem because of abundant population-level genetic and phenotypic variation in the gut inflammatory response. Previous work in our laboratory identified genetically divergent stickleback populations exhibiting differences in intestinal neutrophil activity. We took advantage of this diversity to genetically map variation in an emblematic element of gut inflammation - intestinal neutrophil recruitment - using an F2-intercross mapping framework. We identified two regions of the genome associated with increased intestinal inflammation containing several promising candidate genes. Within these regions we found candidates in the Coagulation/Complement System, NFkB and MAPK pathways along with several genes associated with intestinal diseases and neurological diseases commonly accompanying intestinal inflammation as a secondary symptom. These findings highlight the utility of using naturally genetically diverse ‘evolutionary mutant models’ such as threespine stickleback to better understand interactions among host genetic diversity and microbiome variation in health and disease states.

Animals harbor an array of microbes on and in their bodies which perform essential functions that are fundamental to host health (Fraune and Bosch 2010; Relman 2012; McFall-Ngai *et al.* 2013). Maintaining appropriate host-microbe interactions by facilitating the presence of symbionts and removing pathogens is therefore vital to sustaining health (Bates *et al.* 2006; Blaser and Falkow 2009; Round and Mazmanian 2009; Chung *et al.* 2012; Jostins *et al.* 2012). Interactions between the host immune system and resident microbes are at the center of this relationship (Bates *et al.* 2006; Ley *et al.* 2008; Blaser and Falkow 2009; Round and Mazmanian 2009; Chung *et al.* 2012; Jostins *et al.* 2012; Relman 2012; McFall-Ngai *et al.* 2013). The immune system can promote beneficial microbes that increase host fitness, and failed interactions can result in a persistent inflammatory response, with the immune system chronically responding negatively to resident microbes. This in turn results in diseases such as Ulcerative Colitis and Crohn’s Disease (Eckburg and Relman 2007; Emilsson *et al.* 2008; Graham and Xavier 2013).

The relationship between host immune system and resident microbes is complex. Some microbes cause disease states only in specific host genetic backgrounds or in the presence of other microbes (Casadevall and Pirofski 2000). For example, important work in humans has revealed a strong influence of genetic variation on health outcomes particularly in the context of additional microbiome variation (Dethlefsen *et al.* 2007; Manolio *et al.* 2009; Ko *et al.* 2009; Torkamani *et al.* 2012; Goodrich *et al.* 2014). In addition, these host-microbe interactions can be mediated by internal environmental conditions such as stress physiology (Lupp *et al.* 2007; Alverdy and Luo 2017; Wagner Mackenzie *et al.* 2017) and external conditions such as diet (Hildebrandt *et al.* 2009; Albenberg and Wu 2014; Voreades *et al.* 2014; Singh *et al.* 2017). As such, variation in host-associated microbiomes can productively be considered a quantitative trait.

What is needed are studies that can link quantifiable microbe-induced differences in immune response to host genomic loci and genetic variants. One way to quantify the inflammatory response is through assessment of neutrophils, specialized white blood cells that are recruited during an inflammatory response (Bradley *et al.* 1982; Renshaw *et al.* 2006; Kumar and Sharma 2010; Mantovani *et al.* 2011; Kolaczkowska and Kubes 2013). These cells exist throughout the body and are recruited from the blood stream to sites of inflammation, including the gut (Borregaard 2010; Fournier and Parkos 2012; Wera *et al.* 2016). While intestinal neutrophil recruitment often occurs due to the presence of pathogens, resulting from acute inflammation, such recruitment can also occur chronically due to aberrant interactions between the immune system and the gut microbiota (Foell *et al.* 2003; Wera *et al.* 2016; Mortaz *et al.* 2018; Rosales 2018; Murdoch and Rawls 2019).

Genomic regions that underlie these complex inflammatory phenotypes associated with neutrophil variation can be identified using genetic mapping in model organisms through the use of mutational screens (Musani *et al.* 2006; Hillhouse *et al.* 2011; Leach *et al.* 2012; Uddin *et al.* 2011; Chen *et al.* 2016; Barry *et al.* 2018). Because of the complex interplay of genetics, microbes and environment, it is also essential to develop outbred mutant models tractable for genetic mapping of *natural* genetic variants influencing complex phenotypes such as inflammation (Albertson *et al.* 2009; Gasch *et al.* 2016). Here, we use the threespine stickleback fish (*Gasterosteus aculeatus*) as such an outbred ‘evolutionary mutant model’ (Albertson *et al.* 2009) to study just such complex disease traits.

This small teleost fish is found throughout the arctic in a wide range of environments including freshwater and oceanic habitats, resulting in exceptional degrees of within -and among- population genetic and phenotypic variation for countless traits (Bell and Foster 1994; Colosimo *et al.* 2004; Cresko *et al.* 2004, 2007; Hohenlohe *et al.* 2010; Glazer *et al.* 2015; Lescak and Milligan-Mhyre 2017). Notably, there are multiple high quality genome assemblies from disparate populations (Jones *et al.* 2012; Peichel *et al.* 2017) and the large clutch sizes of stickleback provide ample family sizes for QTL mapping (Colosimo *et al.* 2004; Cresko *et al.* 2004; Kimmel *et al.* 2012; Miller *et al.* 2014; Glazer *et al.* 2015; Greenwood *et al.* 2015; Peichel and Marques 2017). By using threespine stickleback lines originating from genetically diverse populations with distinct ecological and evolutionary histories we are able to map natural genetic variants thus allowing us to identify the types of variants likely underlying this complex phenotype in the human population (Albertson *et al.* 2009).

Previous work in our laboratory described phenotypic variation between freshwater and oceanic ecotype inflammatory responses, with oceanic individuals responding more robustly to the presence of microbes measured by an increase in intestinal neutrophil accumulation and changes in gene expression (Milligan-Myhre *et al.* 2016; Small *et al.* 2017). These findings identified a potential role of host genetic variation on differences in intestinal inflammation and the response to the presence of microbes across populations. We set out to map natural genetic variants associated with differences in intestinal neutrophil density using an F2-intercross genetic mapping study in threespine stickleback. We used these data to identify genomic regions that, when combined with previously published gene expression data from juvenile guts in the parental populations (Small *et al.* 2017), identified a concordant list of candidate genes involved in host immunity. Surprisingly, we also found several genes with characterized functions in neurological diseases known to include intestinal inflammation as a secondary symptom. These findings have broader impacts in elucidating roles of natural genetic variation in chronic intestinal inflammation and provide further evidence of a strong link between intestinal health and systemic inflammation.

## Materials and Methods

### Husbandry and experimental design

We generated an F2 mapping cross of threespine stickleback derived from wild caught Alaskan populations previously maintained in the laboratory for at least ten generations. An F1 line was generated by *in vitro* fertilization of parents derived from two distinct Alaskan populations including a male from the freshwater population Boot Lake (N 61.7167, W 149.1167) and a female from the anadromous population Rabbit Slough (N 61.5595, W149.2583). An F2 family (n = 64) was produced intercrossing F1 siblings. Fertilized eggs were incubated overnight in one-micrometer filter sterilized antibiotic embryo media containing 100 mg/mL Ampicillin, 50 mg/mL Kanamycin, and 8 mg/mL Amphotericin (Milligan-Myhre *et al.* 2016; Small *et al.* 2017). The next day embryos were surface sterilized using 0.003% bleach solution and 0.2% Polyvinylpyrolidone-iodine (PVP-I) solution (Western Chemical Inc.) following protocols described by Small *et al.* 2017. Fish were raised in sterile stickleback embryo media until 9 days post fertilization (dpf) when they were moved to a 9.5 liter tank exposing them to the microbes present in the Cresko Lab fish facility. At this time, fish were housed under “summer” conditions of 16 hr of daylight and 8 hr of night where they were fed 2 mL of hatched brine shrimp naupli and fry food (Ziegler AP100 larval food) designed to mimic the diet of wild stickleback. Water temperature was maintained at 20° with salinity at 4 parts per thousand (PPT) on a recirculating system. For the entirety of the experiment, all F2 siblings we cohoused removing environmental variation as a possible contributor to phenotypic variation. At 14 dpf, fish were euthanized with MS222, following IACUC approved methods described by Cresko *et al.* 2004. Fish were imaged for standard length measurements, tail-clipped, and fixed overnight in 4% paraformaldehyde (PFA) at room temperature then moved to 4° for long term storage.

### MPO staining and phenotyping

Whole fish were stained using the Sigma Myeloperoxidase kit (Sigma, 390A-1KT, St Louis, MO, USA), which preferentially stains neutrophils. Fish were then embedded in paraffin, and their bodies were cross-sectioned in 7-micrometer sections from just posterior to the gill rakers to the urogenital opening. Every 10^th^ section beginning posterior to the gill rakers was imaged. Neutrophils were counted twice in each imaged section. If the counts did not agree, the section was counted a third time. Average neutrophils per section were then calculated and tested for association with standard length and sex using R v3.4.1 (R Core Team 2017).

### Statistical analysis of phenotypic variation

**Figure 1:**
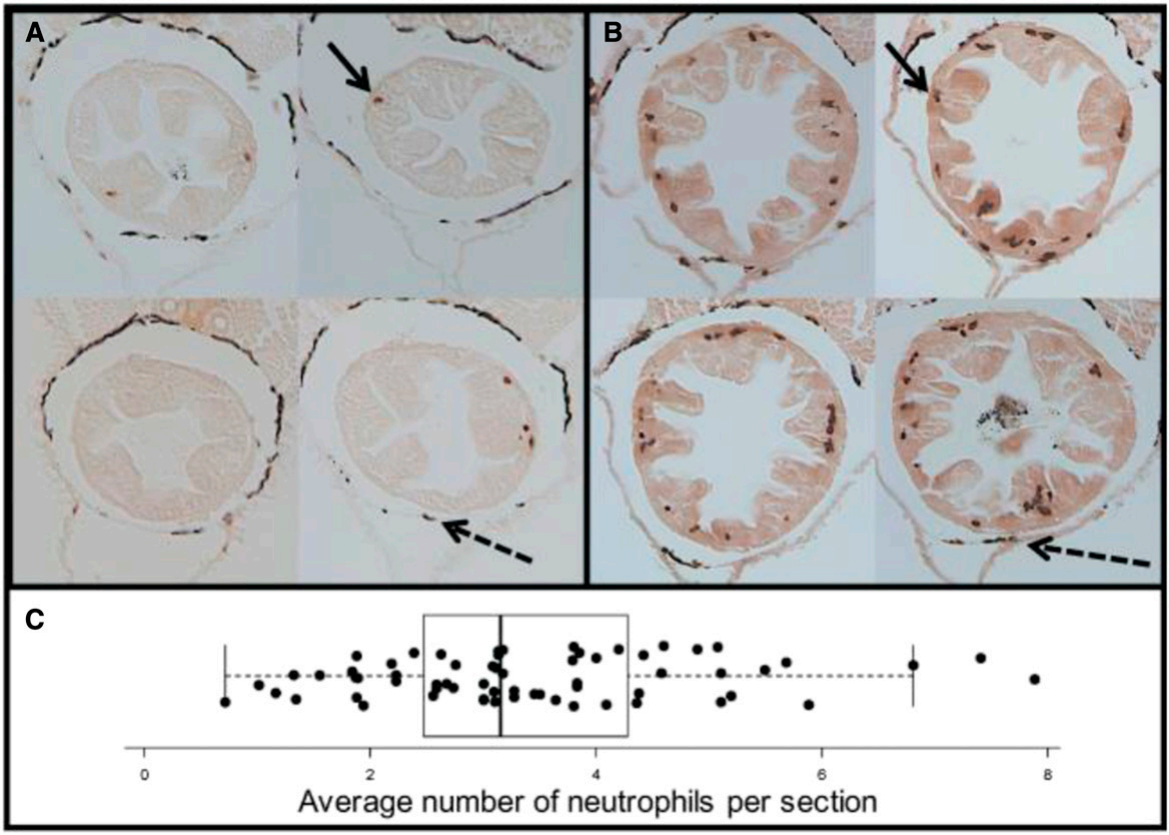
Phenotypic Variation of Intestinal Neutrophil Recruitment. (A) Gut sections of a 14 dpf fish with a low average neutrophil count per section. (B) Gut sections of a 14 dpf fish with a high average neutrophil count per section. (C) F2 Phenotypic distribution of variation of intestinal neutrophil recruitment.

Intestinal neutrophil abundance in 14 days post fertilization (dpf) F2s was distributed roughly normally, with full siblings ranging from less than one neutrophil per section on average to over seven neutrophils per section (Figure 1). This variation was shown to be independent of sex (t = 1.48; *P* = 0.14) (Figure S1), as males and females exhibited similar distributions of average intestinal neutrophils. To determine if differences in neutrophil density correlated with minor changes in developmental timing and growth rate, we used standard length which is a common metric used for fish. In doing this, we found that neutrophil abundance is correlated with standard length, with larger fish exhibiting higher average neutrophil density (R^2^= 0.14; *P* = 0.0013) (Figure S2). This measurement of standard length was additionally correlated with gut size, with larger fish exhibiting larger guts (R^2^ = 0.14; *P =* 0.001) (Figure S3). However, there is no direct relationship between gut size and neutrophil density (R^2^ = -0.016; *P =* 0.9) (Figure S4). We therefore concluded that differences in developmental timing, measured by standard length, should be included as a covariate for subsequent analyses. To account for size as a covariate, we calculated residuals from the regression of neutrophil density and standard length, to be included as trait values in our QTL mapping.

### DNA isolation and sex determination

Tail samples were flash frozen in liquid nitrogen and stored at -80°. DNA was extracted using the Qiagen DNeasy Blood and Tissue Kit (Qiagen, Valencia, CA, USA). DNA was then quantified using the Qubit fluorometer broad range kit. Individual sex phenotypes were determined using PCR amplification of a sex specific region of the genome using the GA1 primer pair (Griffiths 2000), and males were identified by the presence of the male-specific amplicon.

### Genotyping of parents and progeny

Genomic DNA from each F1 parent and F2 offspring was standardized to 10 ng/uL and digested with the endonuclease SbfI-HF (NEB), and RAD-seq libraries were generated using protocols established by the Cresko Laboratory (Hohenlohe *et al.* 2010; Baird *et al.* 2008; Etter *et al.* 2011). In some progeny samples, DNA concentrations fell below the 10 ng/uL threshold, but all samples with at least 100 ng of DNA were used. Uniquely barcoded samples were then sequenced in one lane on the Illumina HiSeq 4000 to obtain single end 150 bp reads. To improve coverage, the lane was re-run through a second round of sequencing on the HiSeq 4000. Raw sequence data were demulitplexed by barcode and filtered using the process_radtags program in the Stacks suite v1.48 (Catchen *et al.* 2011; Catchen *et al.* 2013). Together these sequencing lanes yielded 799,824,397 reads with 708,390,956 reads retained, averaging 3,873,856 reads retained per individual. Reads were then aligned using GSNAP (Wu and Watanabe 2005) to the stickleback reference genome from Ensembl (version 80), allowing for seven maximum mismatches and a terminal threshold of ten. These flags were implemented to avoid soft clipping and spurious alignment of short reads. Genotypes were called using the ref_map pipeline of the Stacks suite.

### QTL mapping

Genotype calls were concatenated in a VCF file generated using the populations package in Stacks (Catchen *et al.* 2011; Catchen *et al.* 2013). Filtering was then performed using VCFtools (Danecek *et al.* 2011). For a marker to be included in the analysis, it was required to have a genotype call in at least 50% of the progeny. MapMaker files were then generated via text manipulation using custom scripts to include all 18,394 SNPs from the filtered VCF file. QTL mapping was then performed using the r/QTL *scanone* function with Haley-Knott regression (Broman and Sen 2009; Broman *et al.* 2003). Percent variance explained was estimated using the highest LOD SNP for each retained peak using an additive model implemented in the *fitqtl* function allowing for an error probability of 5%. To include growth rate as a covariate, QTL mapping was also performed on residuals calculated from a regression of neutrophils per section on standard length. Genomic regions were further analyzed using the raw neutrophil count data if QTL were preserved in both the raw and residual data analyses. To account for potential false positives, r/QTL was re-run with phenotypes randomly assigned to genotypes.

### Functional assignments of associated SNPs

To identify differentially expressed genes, we data-mined RNA-seq differential expression data published by Small *et al.* 2017. These differential expression analyses were a particularly strong choice in identifying candidate genes in our F2s, as these data were generated from the same populations as the grandparents of our F2s. Additionally, these differential expression analyses were performed on 14 dpf guts, the same timepoint as our F2s. To assign function to genes within each of our associated SNP boundaries, we used GeneCards database v4.8.1 Build8 based on assumed homology to human genes. While the use of human gene function is common practice to assign function to model organism orthologs, it should be noted that this is not always accurate and functional work is needed to assign gene function with certainty.

### Data availability

Sequencing data are publicly available in the Sequencing Read Archive (SRA) under project ID: PRJNA591028 Supplemental material available at figshare: https://doi.org/10.25387/g3.11367854.

## Results

### Several genomic regions associated with increased intestinal neutrophil density independent of stickleback size

**Figure 2:**
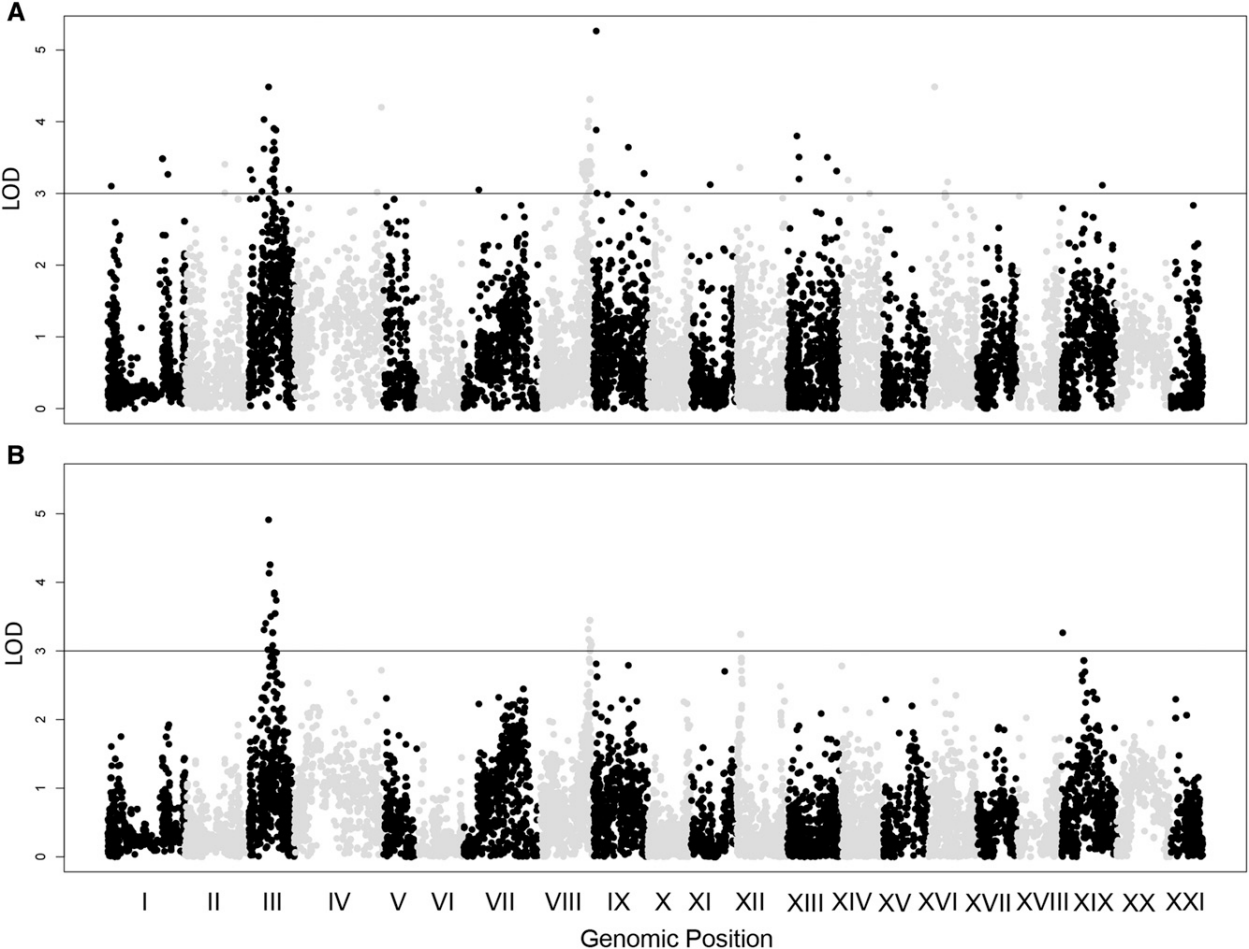
QTL Maps of Average Neutrophil Counts. (A) Raw neutrophil count data (B) Residual data including standard length as a covariate. Linkage Groups alternate in color Black/Gray. Horizontal line indicates a LOD cutoff of 3.

We identified 18,394 SNPs to be used for QTL mapping. Using raw intestinal neutrophil densities (number of neutrophils per section), we detected 13 linkage groups (LG) with QTL associated with variation in neutrophil density (LOD > 3) (Figure 2; Table 1). These included LG1, LG2, LG3, LG4, LG7, LG8, LG9, LG11, LG12, LG13, LG14, LG16, and LG19. To disentangle these findings from standard length and subsequently avoid mapping genes associated with developmental rate or growth, we mapped the residuals of neutrophil number included in a linear model with standard length as a covariate (Figure 2; Table 1). The number of regions of the genome likely associated with intestinal neutrophil recruitment was greatly reduced when mapping the residuals. QTL retained included pile-ups on LG3, LG8, and LG12. A new peak on LG19 also appeared in mapping the residuals, but this was removed as it was not conserved between the raw and residual maps.

**Table 1:**
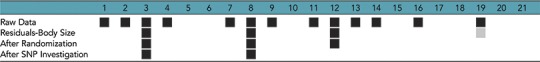
QTL identified in each Linkage Group (1-21) at each filter step. Black indicates a retained association. Gray indicates a new association on the same Linkage Group

In some instances, low coverage or missing genotypes can falsely inflate LOD scores of individual SNPS generating false positives. To evaluate our data for false positives, we randomized the raw phenotype data and the residuals with respect to the F2 genotypes and re-ran r/QTL. This method is similar to large scale permutation tests used to designate a significant alpha value, but can also indicate regions where low coverage in specific SNPs are generating false positives. In our original runs, high-LOD regions were concentrated in pileups (Figure 2), indicating the type of associative signal expected from linkage mapping. This is in stark contrast to our randomized dataset, with high-LOD SNPs scattered sparsely across the genome (Figure S5). Most importantly, analysis of the randomized datasets revealed very little overlap with our original runs, providing support that low coverage was not generating false positives (Figure S5). The only overlapping signal between the original and randomized data were the pattern of two pileups on LG1, which ultimately fell below the significance threshold upon mapping the residuals, further suggesting a false positive in this region.

To test if low coverage in a few SNPS could be generating false positives, we assessed phenotype distributions for each genotype in the SNPs with LOD > 3, in the regions with retained signal in both the raw and residual maps. On LG12 we found evidence of false positives, with neutrophil densities spanning similar phenotypic space regardless of genotype (Figure S6), with heterozygous and homozygous siblings exhibiting similar average neutrophil densities (t = -1.11; *P =* 0.28; df = 34.7, t = -1.11; *P =* 0.28; df = 34.7 and t = -1.30; *P* = 0.20; df = 43.4). In this case, one parent was a homozygote while the other was a heterozygote so there are only two genotypes in the progeny (homo and het) (Figure S6).

**Figure 3:**
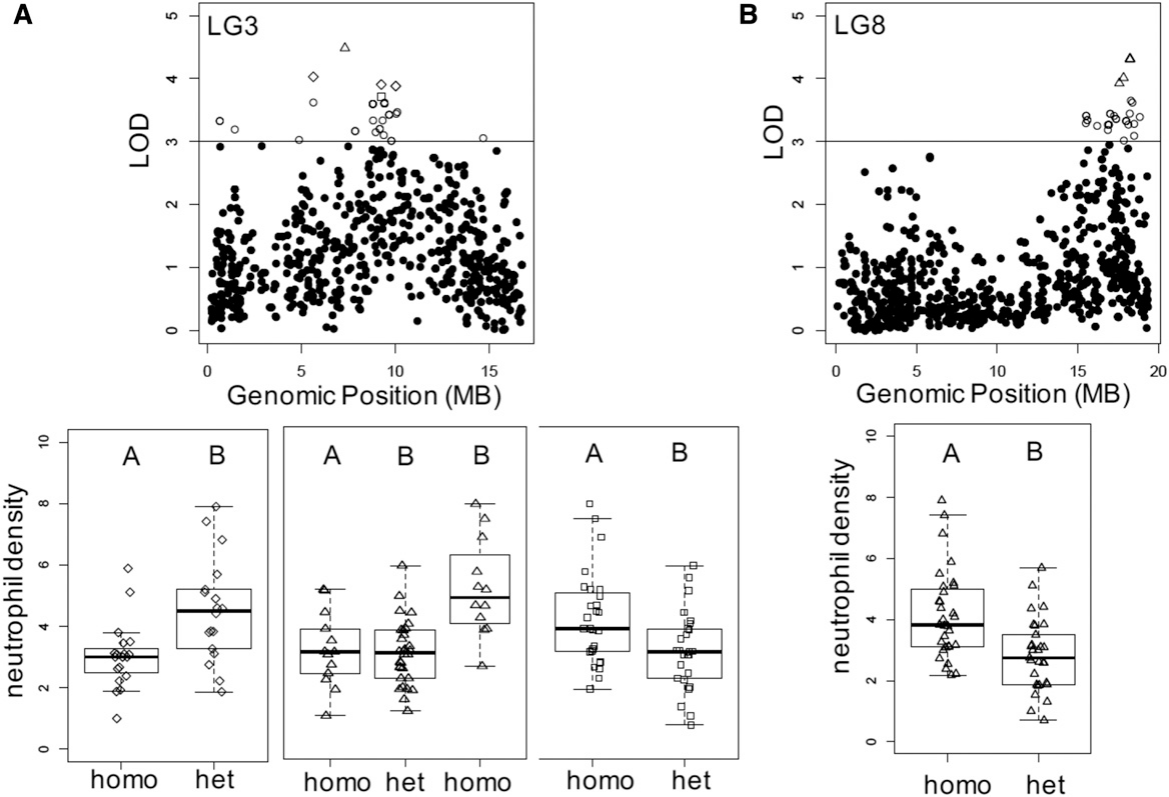
Phenotype Distributions by Genotype. (A) Zoomed in view of LG3 Manhattan plot of raw QTL data. Non-circular shapes indicate SNPs with the highest LOD scores; shapes correspond to boxplots below. (B) Zoomed in view of LG8 Manhattan plot of raw QTL data. Non-circular shapes indicate SNPs with the highest LOD scores; shapes correspond to boxplots below. Relationships between homozygotes and heterozygotes from multiple SNPs are concatenated into single boxplots when they exhibit the same pattern. In some instances, a homozygous parent was crossed to a heterozygous parent yielding two genotypic offspring groups heterozygotes (het) and homozygotes (homo). In other cases, two heterozygous parents were crossed yielding three offspring groups one het group and to homo groups. Letters A and B above the boxplots indicate a statistically significant difference *P* < 0.05.

At the remaining genomic loci with SNPs exhibiting LOD > 3, on LG3 and LG8, all testing suggested true associations of SNPs with differences in neutrophil density. To determine the percent variance explained by genotype we assessed the highest LOD SNP from LG3 and LG8 in an additive model using *fitqtl*. This analysis indicated 15.84% variance and 15.77% variance explained by the genomic variants on LG3 and LG8 respectively. Given the careful control of environmental influence in our experimental design, this was not surprising. However, given the modest sample size of our F2 family, this likely underplays the total influence of genetics on intestinal neutrophil density. On LG3 we subsampled five SNPs exhibiting the highest LOD scores in the pile-up. Three SNPS exhibited a similar pattern: with homozygotes exhibiting a significant decrease in neutrophil density compared to their heterozygous siblings (Table 2; Figure 3; Figure S7). The fourth SNP exhibited the opposite pattern with homozygous siblings exhibiting a significant increase in neutrophil density compared to heterozygous siblings (Table 2; Figure 3; Figure S7). Each of these SNPs resulted from a homozygous and a heterozygous parental genotype cross, generating only two offspring groups. The fifth SNP was generated from two heterozygous parents and therefore was split into three groups: two homozygous groups (AA/TT) and a heterozygous group (AT) (Figure S7). At this locus, fish homozygous for the A allele had a significantly higher average neutrophil density than either other genotypic group while individuals with with genotypes AT or TT did not differ significantly (Table 2; Figure 3; Figure S7). On LG8 we again subsampled the five SNPS with the highest LOD scores which encompassed four RAD markers. In all cases homozygous siblings had a significantly higher neutrophil density than heterozygous siblings (Table 2; Figure 3; Figure S7).

**Table 2:** Phenotypic Distribution summary by SNP

Location*^a^*	Mean Neutrophil Density Homozygote #1	Mean Neutrophil Density Heterozygote	Mean Neutrophil Density Homozygote #2	t*^b^*	df*^c^*	*P*
**LG3**						
5,649,237	3.04	4.52	NA	−3.15	29.01	0.004
9,266,284	4.18	3.13	NA	2.72	50.06	0.009
9,266,333	3.13	4.18	NA	−2.67	49.70	0.010
7,331,556	3.16 (TT)	3.06 (AT)	5.15 (AA)	TT/AT: 0.27049 TT/AA: -3.5077 AT/AA: -4.2347	TT/AT: 20.16 TT/AA: 20.68 AT/AA: 15.08	TT/AT: 0.79 TT/AA: 0.002 AT/AA: 0.0007
10,037,540	3.06	4.66	NA	−2.99	23.00	0.006
**LG8**						
17,581,467	4.07	2.96	NA	3.09	53.90	0.003
17,842,407	4.20	2.78	NA	4.02	52.98	0.0002
18,229,015	4.16	2.84	NA	3.87	56.93	0.0003
18,229,073	4.16	2.84	NA	3.87	56.93	0.0003
18,268,403	4.30	3.06	NA	3.28	46.92	0.002

a Location in base pairs. Reference Genome v80.

b Welch Two Sample t-test statistic.

c Degrees of freedom.

To demarcate genomic intervals of interest based on our retained QTL, we defined boundaries using the outside flanking SNPs LOD < 3 (Table S3). In LG3, we identified 11 SNP clusters LOD > 3, including one marker with a score below the initial threshold of LOD > 3. We chose to include this locus, however, as the LOD score of the residual was one of the highest (LOD = 4.13) and due to linkage, these pileups are used to identify fairly large genomic regions of association. In LG8 we identified 17 such regions (Table S3).

### Immune pathways and disease genes associated with increased intestinal neutrophil density

To ascertain biological relevance of each of our QTL we used the threespine stickleback genome annotation to compile a complete list of candidate genes associated with each genomic interval. Within these boundaries we assembled a complete gene list and identified those with known functions and if available assigned directionality of differential expression from the parental populations (Small *et al.* 2017). Within these intervals we identified several gene groups on interest including members of the Coagulation/Complement Cascade, Mitogen-Activated-Protein Kinase (MAPK) pathway, Extracellular Signal-Reduced Kinase (ERK), the Nuclear factor kappa-light-chain-enhancer of activated B cells (NfKB) immune pathway, and the maintenance of tight junctions (Table 3).

**Table 3:** Candidate Gene Summary

Group	Ensembl ID	Gene	Distance from SNP*^a^* (bp)	Immune Pathways	Disease Associations
LG3	ENSGACG00000014756	*f3*	37,324	Coagulation Cascade	—
LG3	ENSGACG00000015273	*map2k2b*	84,711	MAPK/ERK	—
LG3	ENSGACG00000015282	*tcf3a*	58,000	MAPK/ERK	—
LG3	ENSGACG00000015301	*unc13a*	15,909	—	ALS
LG3	ENSGACG00000015386	*epha4b*	25,437	MAPK/ERK	—
LG3	ENSGACG00000015719	*cldn18*	10,244	Tight Junctions	—
LG3	ENSGACG00000016028	*ripk2*	6,127	NFkB	—
LG3	ENSGACG00000016066	*tblxr1b*	16,774	—	Autism
LG3	ENSGACG00000016121	*rgs1*	1,892	—	Celiac Disease
LG3	ENSGACG00000016159	*pik3R3*	63,634	MAPK/ERK	—
LG3	ENSGACG00000016189	*scp2a*	0	—	Wheat Allergy
LG3	ENSGACG00000016212	*angptl3*	5,349	MAPK/ERK	—
LG3	ENSGACG00000016323	*c8b*	972	Complement Cascade	—
LG3	ENSGACG00000016338	*c8a*	0	Complement Cascade	—
LG8	ENSGACG00000012681	*pias4a*	68,402	NFkB	—
LG8	ENSGACG00000012686	*map2k2a*	58,202	MAPK/ERK	—
LG8	ENSGACG00000012737	*tcf3b*	5,424	MAPK/ERK	—
LG8	ENSGACG00000013097	*borcs8*	19.084	MAPK/ERK	—
LG8	ENSGACG00000013123	*ncan*	0	MAPK/ERK	—
LG8	ENSGACG00000013599	*camk4*	6,656	MAPK/ERK	—
LG8	ENSGACG00000013618	*gadd45bb*	5,900	MAPK/ERK; NFkB	—
LG8	ENSGACG00000013753	*gabrb3*	21,271	—	Autism
LG8	ENSGACG00000013771	*gabrg3*	0	—	Autism
LG8	ENSGACG00000014026	*cldn34*	17,633	Tight Junctions	—
LG8	ENSGACG00000014029	*zgc:153311*	15,831	Tight Junctions	—
LG8	ENSGACG00000014071	*nek1*	80,789	—	ALS
LG8	ENSGACG00000014199	*sgcb*	0	—	Muscular Dystrophy

a indicates closest SNP LOD > 3.

The first group of genes were involved in the Coagulation/Complement Cascade, a pathway also enriched for genes differentially expressed between oceanic and freshwater stickleback families (Small *et al.* 2017). These included *f3*, *c8a*, and *c8b* on LG3 (Table 3). On LG3 one marker was located within the 12^th^ exon of *c8a* (Table S4). A second group included members of the ERK signaling and MAPK pathways. Interestingly, the MAPK Pathway was also enriched for genes sensitive to the presence of microbes in freshwater families (Small *et al.* 2017). Five of these genes were on LG3: *map2k2b*, *epha4b*, *pik3R3*, *angptl3*, and *tcf3a*; six of these genes were on LG8: *borcs8*, *ncan*, *camk4*, *gadd45bb*, *map2k2a* and *tcf3b* (Table 3). None of these genes exhibited differential expression patterns (Small *et al.* 2017) nor were any markers located within the coding region of these genes, but the identification of two co-orthologous pairs (LG3: *map2k2b* and *tcf3a*; LG8: *map2k2a* and *tcf3b*) in addition to the number of genes in this pathway is highly suggestive of a strong association between MAPK/ERK signaling and intestinal inflammation. Another immune pathway associated with several candidate genes was the NfKB immune pathway. This included *ripk2* - an activator of NFkB - on LG3 (Yin *et al.* 2010) and *pias4a* and *gadd45bb* on LG8 (Table 3) (Wang *et al.* 2005; Xu *et al.* 2016).

We also identified several gene candidates associated with human intestinal diseases. This included three genes involved in the formation and maintenance of tight junctions as well as those with direct ties to wheat allergies and Celiac Disease including tight junction genes *cldn18* on LG3 and *cldn34* and *zgc:153311* on LG8 (Table 3) and *rgs1* and *scp2a* also on LG3, which contained three markers within intronic regions (Table 3; Table S4). Interestingly, *cldn18* and *cldn34* were both upregulated in oceanic compared to freshwater families (Table S4) (Small *et al.* 2017).

Finally, we identified several human disease genes associated with neurological/neuromuscular syndromes that have secondary symptoms relating to intestinal inflammation. This included two genes associated with Amyotrophic Lateral Sclerosis (ALS): *unc13a* on LG3 (Diekstra *et al.* 2012) and *nek1* on LG8 (Kenna *et al.* 2016), three genes associated with Autism Spectrum Disorder (ASD): *tblxr1b* on LG3 and *gabrb3* and *gabrg3* on LG8, and one disease gene associated with muscular dystrophy, *sgcb*, on LG8. These finding suggest potential genetic links between neurological disease and intestinal inflammation.

## Discussion

To our knowledge this is the first study in which an F2 intercross mapping framework has been used to identify genomic loci underlying a complex immune trait. The identification of multiple genomic loci significantly associated with variation in neutrophil density suggests several genes throughout the genome contribute to this complex phenotype. Our results highlight several genomic loci with relatively large effects contributing specifically to intestinal neutrophil recruitment. The persistence of high LOD SNP pile-ups on LG3 and LG8, after accounting for standard length as a covariate and testing for false positives, argue for the biological relevance of the QTL detected in this analysis. However, the modest size of this single family of F2 progeny (n = 64) means that other genomic regions of small effect would likely have gone undetected. Although other genes of small effect with bearing on the observed variation in intestinal neutrophil activity may have been missed, we can be confident in our identification of several genomic intervals on LG3, and LG8 with strong associations to intestinal neutrophil activity. It is important to note that this study does not suggest causality of any one of these SNPs individually. Instead these data are highly suggestive of candidate genomic regions and specific genes that warrant further investigation into their ties to intestinal inflammation.

More specifically, the large number of immune genes identified in the associated genomic intervals is not an unexpected result. The Coagulation/Complement Cascade, MAPK, ERK, and NfkB pathways are all essential immune pathways activated early in development and play roles in the regulation of the inflammatory response (Kurosawa *et al.* 2000; Markiewski and Lambris 2007; Liu *et al.* 2007; Dev *et al.* 2011; Arthur and Ley 2013; Liu *et al.* 2017). However, the identification of three co-orthologous pairs was particularly interesting. In the first pair, *c8a* and *c8b* were both found in the same genomic interval with the associated SNP located within *c8a*. These proteins both function as a part of the Complement Cascade, a pathway enriched for differentially expressed genes between oceanic and freshwater stickleback (Small *et al.* 2017). Together, these proteins initiate membrane penetration and coordinate the formation and insertion of the membrane attack complex (MAC) into the bilayer to facilitate lysis (Bubeck *et al.* 2011). The other co-orthologous pairs *map2k2a/map2k2b* and *tcf3a/tcf3b* are also excellent candidates for impacting the inflammatory response as *tcf3* has been shown to play a role in the regulation of B cell maturation and *map2k2* is an activator of MAPK. These pairs are of particular interest, however, as they were found on separate linkage groups with *map2k2b/tcf3a* on LG3 and *map2k2a/tcf3b* on LG8.

The identification of several genes involved in human intestinal disease via the formation and regulation of tight junctions was also intriguing. Tight junctions are extremely important in regulating intestinal permeability and the intestinal immune response, and have been tied to many intestinal diseases including IBD, Celiac Disease, and Type 1 Diabetes (Visser *et al.* 2009; Castoldi *et al.* 2015; Lee 2015; König *et al.* 2016) as well as autoimmune disease (Arrieta *et al.* 2006; Mu *et al.* 2017). Mutations in tight junctions have also been associated with leaky gut with direct ties to the microbiome with probiotic treatments resulting in the increase of tight junction formations and the reversal of leaky gut (Mu *et al.* 2017). Impact on tight junction could easily explain differences in the intestinal inflammatory response. Further tests assessing differences in the abundance and functionality of tight junctions in oceanic and freshwater stickleback are needed to understand these potential contributions to observed differences in intestinal neutrophil activity. Importantly, coinciding with this group were two additional disease genes associated with wheat allergies and Celiac Disease, *rgs1* and *scp2a*, which contained three markers within intronic regions (Table 3; Table S4).

Lastly, the identification of variants within neurological and neuromuscular disease genes provides potential links between intestinal and neurological health. Our findings included variants associated with known disease genes impacting ALS, Autism, and Muscular Dystrophy. All of these diseases are of particular interest to those studying intestinal inflammation, as individuals who exhibit them often report symptoms consistent with colitis and other types on intestinal inflammation at greater rates; and in many cases targeted treatments of the microbiome have been successful in alleviating or slowing progression of symptoms (Nowak *et al.* 1982; Bellini *et al.* 2006; Kaneko and Hachiya 2006; Fang 2016; Lo Cascio *et al.* 2016; Rowin *et al.* 2017; Hughes *et al.* 2018; Opazo *et al.* 2018; Patusco and Ziegler 2018; Spielman *et al.* 2018; Wright *et al.* 2018). How the genetics of these complex diseases are tied to intestinal health is still an unresolved problem and requires further mapping of inflammatory phenotypes, but these targets provide a strong starting point to investigate broader implications on intestinal inflammation on systemic health.

## Conclusions

This study provides a strong example of the power of threespine stickleback as a model for mapping natural variants contributing to genetically complex phenotypes relevant to human disease. To expand upon these findings, we can use the stickleback system to map other related immune system phenotypes and expand studies of inflammation to other tissue types. Though this study does not include functional testing of candidate loci, these findings additionally provide potential targets for functional testing using CRISPR-Cas9 genome editing to connect systems-level genetic links between tight junction, neurological disease, and intestinal health. Because of the amenability of stickleback for gnotobiotic studies, these genetic approaches will be particularly useful to manipulate both host genes and microbiomes simultaneously to perform functional tests not possible in other organisms. Our findings have broader impacts in elucidating the complex roles of natural genetic variation in chronic intestinal inflammation and provide further evidence of a strong link between intestinal health and systemic health.
